# Creep behavior of reinforced concrete-filled steel tubular columns under axial compression

**DOI:** 10.1371/journal.pone.0255603

**Published:** 2021-09-20

**Authors:** Ni Zhang, Chenyang Zheng, Qingwei Sun

**Affiliations:** School of Civil Engineering, Liaoning Technical University, Fuxin, China; China University of Mining and Technology, CHINA

## Abstract

The reinforced concrete-filled steel tube (RCFST) column solves several of the problems of the concrete-filled steel tube (CFST) column in practical engineering applications. Moreover, RCFST has a simple joint structure, high bearing capacity, good ductility, and superior fire resistance. From a structural safety perspective, designers prioritize the creep performance of CFST members in structural design. Therefore, the creep behavior of RCFST columns should be thoroughly investigated in practical engineering design. To study the influence of the creep behavior of RCFST columns under axial compression, this work analyzed the mechanical behavior of composite columns based on their mechanical characteristics under axial compression and established a creep formula suitable for RCFST columns under axial compression. A creep analysis program was also developed to obtain the creep strain–time curve, and its correctness was verified by existing tests. On this basis, the effects of the main design parameters, such as the stress level, steel ratio, and reinforcement ratio, on the creep behavior were determined and analyzed. The creep of the tested composite columns increased rapidly in the early stages (28 days) of load action; the growth rate was relatively low after 28 days and tended to stabilize after approximately six months. The stress level had the greatest influence on the creep of RCFST columns under axial compression, followed by the steel ratio. The influence of the reinforcement ratio on the creep behavior was less. The results of this study can provide a reference for engineering practice.

## Introduction

The reinforced concrete-filled steel tube (RCFST) column is formed by placing longitudinal steel bars in a steel tube and pouring concrete into the tube. The steel tubes of RCFST columns do not bear longitudinal load directly; thus, they can play a constraining role on the core reinforced concrete in the early stages of member loading. However, the steel tubes of CFST members typically bear longitudinal load in a normal operation. The outer steel tube provides a limited restraint to the core concrete when it reaches the yield. Consequently, the axial compressive bearing capacity and seismic performance of RCFST columns with steel tube constraints are better than those of CFST columns with the same parameters [1–3].

RCFST column constraints simplify the joints and reduce the design and construction difficulties. The fire failure of an external steel tube under longitudinal load is often considered in CFST columns, and the reinforcement in RCFST columns improves their fire resistance. Thus, any fire damage to the steel tube would have little influence on the fire resistance limit of the columns [4–6]. RCFST columns overcome some of the drawbacks of CFST columns in practical applications, with advantages of high bearing capacity, simple joint structure, and good ductility. Owing to its excellent performance, the structure is widely used in super high-rise buildings, large-span space structures, and heavy-duty complex buildings in recent years [7, 8].

During the long-term use of RCFST columns, the concrete in the steel tube undergoes compression creep and the internal force is redistributed between the steel tubes, steel bars, and concrete. The continuous change in the concrete creep affects the strain state of the columns. The creep behavior of concrete is more complicated because the 3D stress state of the concrete is constrained by the external steel tubes. Therefore, the ability to accurately predict the creep behaviors is significant in the application of RCFST columns.

Many studies have been conducted on the creep behavior of CFST columns. Furlong [9] was the first to perform a systematic study on CFST composite structures. The author studied short and long CFST columns under axial compression. Through the creep test of 39 CFST composite members, the author concluded that the bearing capacity of the specimens under intermittent loading was lower than that under continuous loading. However, the effect of an external steel tube on the internal concrete was not considered. Nakai et al. [10] conducted long-term load-bearing tests on concrete-filled circular steel tubes and plain concrete. They compared and analyzed the significant differences in their shrinkage and creep deformation behaviors. The long-term deformation of CFST was found to be much lower than that of plain concrete because of the sealing effect of the steel tube and vertical load.

Terry et al. [11] studied the influence of the creep deformation of concrete-filled circular steel tube specimens and plain concrete specimens under long-term loading (100 days). They applied two types of bonding conditions to the concrete-filled steel tube: one wherein the steel tube and the core concrete were well bonded to achieve the same deformation, and another wherein oil was applied between the steel tube and the core concrete to separate them. The test results showed that the influence of the bonding between the steel tube and core concrete on the shrinkage deformation of core concrete can be ignored. Moreover, the elastic modulus and creep coefficient of each specimen under different factors were lower than the corresponding values of reinforced concrete. Uy et al. [12] conducted a long-term load-bearing test on concrete-filled square steel tubular specimens and found that the influence of concrete shrinkage deformation of the members can be ignored. Ichinose et al. [13] studied the influence of loading methods on the creep of concrete-filled steel tubes. The loading methods included loading on the steel tube, loading on the concrete, and loading on the composite surface of the concrete. They conducted creep tests on eight circular concrete-filled steel tubes for 280 days. Naguib et al. [14] combined the flow rate method with a double power function to predict the creep of concrete-filled steel tubes. On this basis, they proposed a creep model of the CFST members under axial compression, considering the multi-axial stress state of the core concrete and Poisson’s ratio.

In China, Zhong et al. [15] were the first to study the creep behavior of CFST columns and the influence of creep deformation on the ultimate bearing capacity of the members. Their results showed that the creep of concrete-filled steel tubes develops rapidly in the early stages but more gradually in the later stages. Wang et al. [16–20] derived creep calculation formulas for CFST members under eccentric and axial compression. The formulas not only reflected the characteristics of the CFST members under axial and eccentric compression, but also fully considered the influence of various factors on the creep of the composite members. On this basis, they compared the accuracy of different theories in the creep calculation of concrete-filled steel tubes.

Furthermore, to study the creep of concrete-filled circular steel tubular members under axial compression, Wang et al. [16–20] used the micro-prestressing consolidation theory to analyze the effects of concrete strength, curing conditions, temperature, and humidity on the creep behavior of these members. For the creep calculation of flexural CFST members, they derived a creep formula based on the theory of continuous flow, considering factors such as the steel ratio, bending moment size, and time. However, research on the creep behavior of RCFST columns has been limited. Qu and Zhang [21] explored an axial deformation measuring device suitable for creep testing of RCFST through the loading test of three measuring schemes. They analyzed the effects of loading age, reinforcement ratio, steel content, and bond friction. Further, they conducted long-term behavior tests of 16 RCFST short columns under axial compression, and obtained the corresponding long-term deformation curves. Li [22] conducted a theoretical analysis and an experimental study on the creep behavior of RCFST short columns under axial compression. The long-term loading tests of eight RCFST short columns and axial compression tests of two steel tube confined concrete short columns were conducted. The results of the theoretical calculation and test were compared and analyzed.

In this study, based on the mechanical characteristics of RCFST composite columns, their mechanical performance was analyzed, and a calculation formula for the axial compression creep was established. Further, a creep analysis program was developed to calculate and analyze the influence of the main design parameters—such as the stress level, steel ratio, and reinforcement ratio, on the creep performance—to provide a reference for engineering practice.

## Force analysis of initial state

### Binding force in elastic stage

For common RCFST members, the maximum load in the elastic stage is approximately in the range of 70%–80% of the ultimate load. For the most practical projects, the service load is typically in the range of 50%–60% of the ultimate load [23]. Therefore, this study analyzed the creep characteristics of RCFST in the elastic working range only.

**Fig 1:**
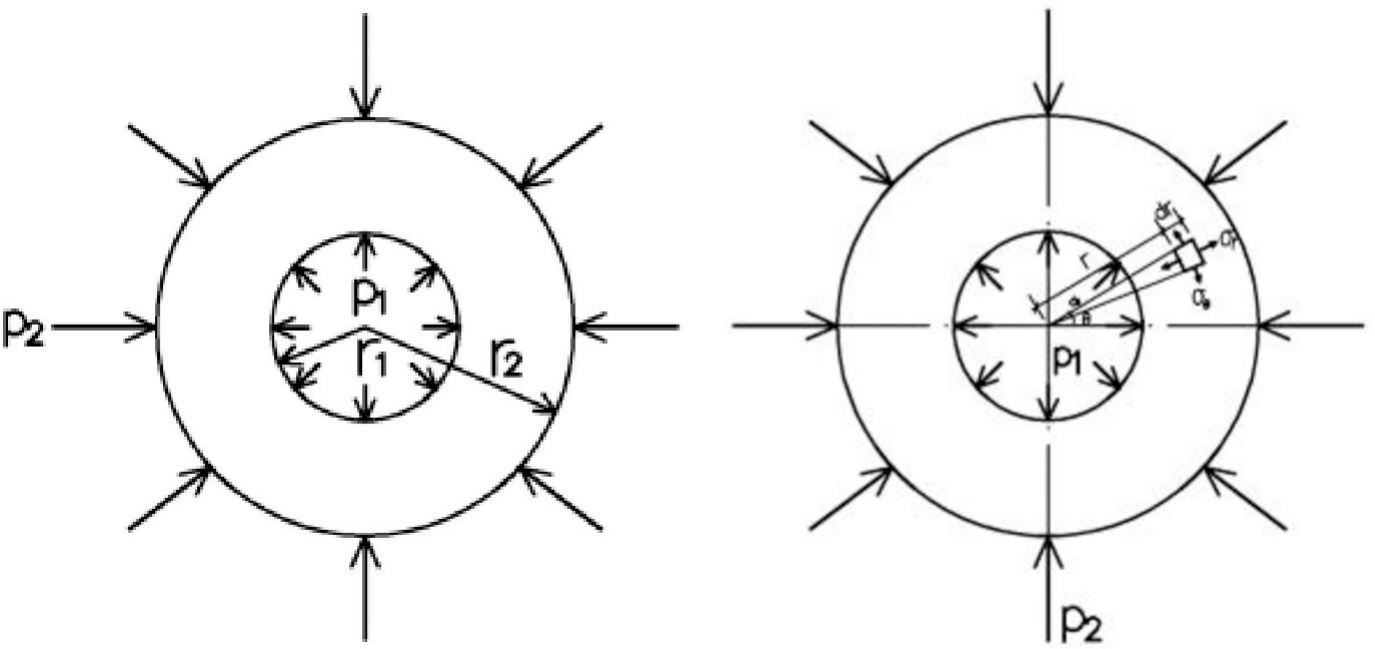
Stress diagram of cylinder.

Lamé’s theory of elasticity [24] is widely used for calculations of thick-walled cylinders. The forces exerted on the cylinder are illustrated in Fig 1.

$$\sigma_{r}=\frac{p_{1}r_{1}^{2}-p_{2}r_{2}^{2}}{r_{2}^{2}-r_{1}^{2}}-\frac{(p_{1}-p_{2})r_{1}^{2}r_{2}^{2}}{r_{2}^{2}-r_{1}^{2}}\cdot \frac{1}{r^{2}}$$ (1)

$$\sigma_{\theta }=\frac{p_{1}r_{1}^{2}-p_{2}r_{2}^{2}}{r_{2}^{2}-r_{1}^{2}}+\frac{(p_{1}-p_{2})r_{1}^{2}r_{2}^{2}}{r_{2}^{2}-r_{1}^{2}}\cdot \frac{1}{r^{2}}$$ (2)

where *σ*_*r*_ and *σ*_*θ*_ are the radial and circumferential stresses at any point on the cylinder wall, respectively. *p*_1_ and *p*_2_ are the internal and external pressures of the cylinder. *r*_1_ and *r*_2_ are the internal and external radii of the cylinder. *r* is the radius at any point on the cylinder.

#### Stress analysis of steel tube

The stress of the steel tube-reinforced concrete section was calculated using Lamé’s formula. *r*_2_ = *r*_*s*_+*t*_*s*_ (*t*_*s*_ is the wall thickness of the steel tube), *p*_2_ = 0, *t*_*s*_/*R*_*s*_<<1, $R_{s}^{2}/\rho^{2}\approx 1$, $r_{s}^{2}/(R_{s}^{2}-r_{s}^{2})=r_{s}^{2}/(2r_{s}t_{s}+t_{s}^{2})\approx r_{s}/2t_{s}$. (*R*_*s*_, *r*_*s*_, and *ρ* are the outer radius, inner radius, and the radius at any point on the steel tube, respectively).

**Fig 2:**
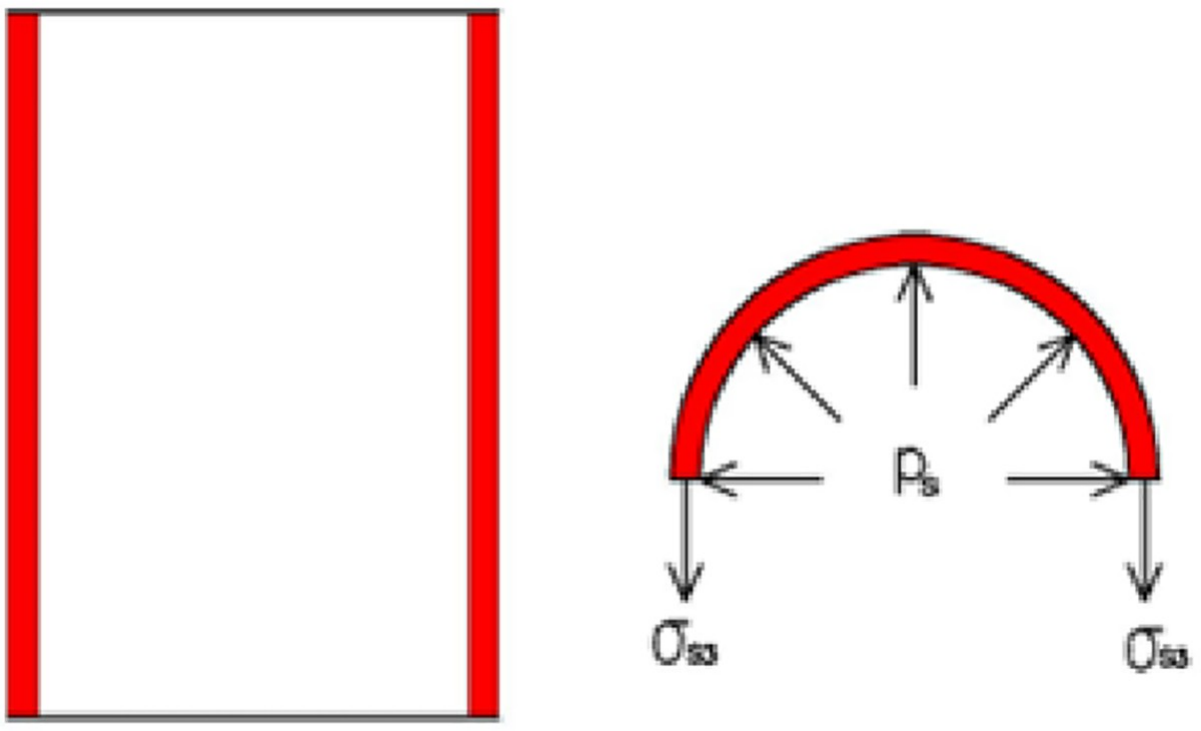
Stress diagram of steel tube.

The circumferential and radial stresses of the steel tube are as follows: $\sigma_{\theta }=\frac{r_{s}}{t_{s}}p_{s}$, *σ*_*r*_ = 0. Here, *p*_*s*_ is the restraint stress of the steel tube. Fig 2 shows the stress of the steel tube.

The radial and circumferential stresses of the steel tube can be expressed as follows:

$$\sigma_{s2}=0,\;\sigma_{s3}=\frac{r_{s}}{t_{s}}p_{s}$$ (3)

The strain of the steel tube can be expressed as follows:

$$\varepsilon_{s3}=\frac{\sigma_{s3}}{E_{s}}$$ (4)

where *E*_*s*_ and *μ*_*s*_ are the elastic modulus and Poisson’s ratio of the steel tube, respectively.

#### Stress analysis of concrete

**Fig 3:**
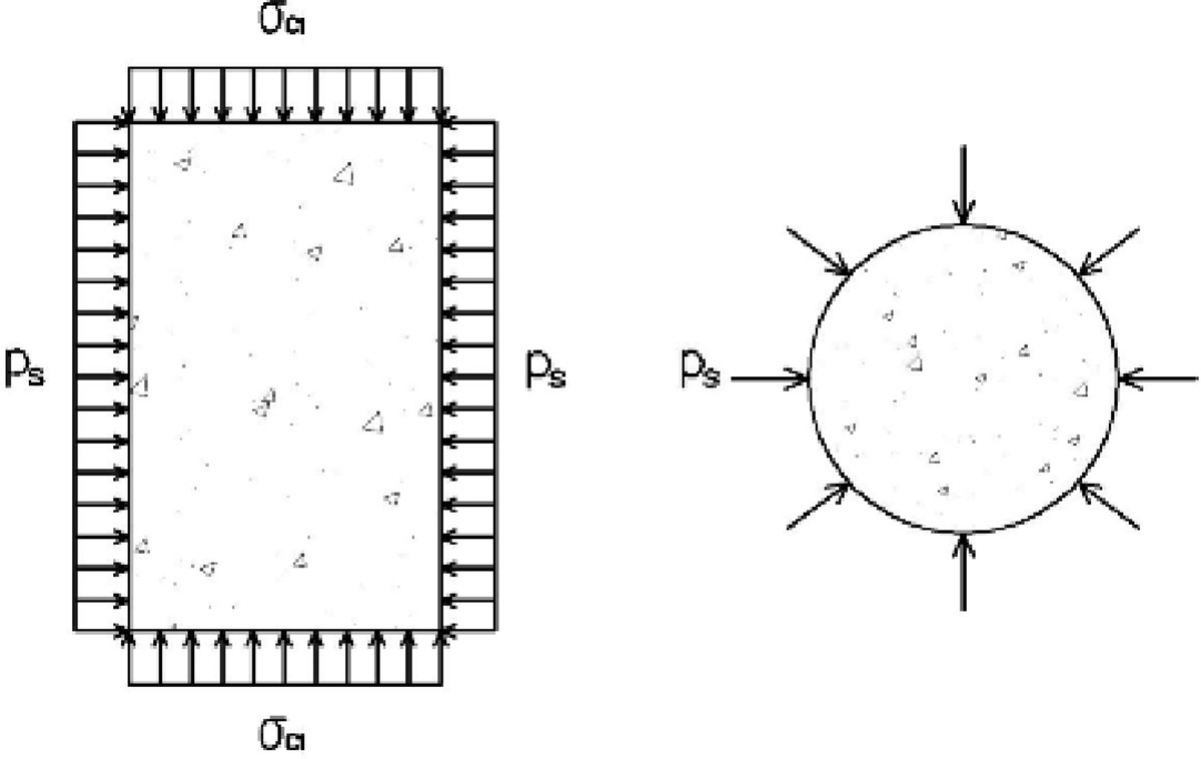
Stress diagram of concrete.

Fig 3 shows the stress of the concrete.

The concrete is in the state of equal lateral stress: *σ*_*c*2_ = *σ*_*c*3_ = *p*_*s*_

According to Hooke’s law, the strain of the concrete can be obtained as follows:

$$\varepsilon_{c1}=\frac{\sigma_{c1}}{E_{c}}-\frac{2\mu_{c}}{E_{c}}p_{s}$$ (5)

$$\varepsilon_{c2}=\frac{p_{s}}{E_{c}}-\frac{\mu_{c}}{E_{c}}(\sigma_{c1}+p_{s})$$ (6)

where *σ*_*c*1_, *E*_*c*_, and *μ*_*c*_ are the longitudinal stress, elastic modulus, and Poisson’s ratio of concrete, respectively.

According to the formula provided by Mao and Wang [25], *μ*_*c*_ varies with the creep:

$$\mu_{c}=F+(0.5-F){\left(\frac{\sigma_{c}}{\left[\sigma_{0}\right]}\right)}^{G}$$ (7)

$$[\sigma_{0}]=f_{ck}+5.5f_{ck}{\left(\frac{p_{0}}{f_{ck}}\right)}^{0.91}$$ (8)

$$p_{0}=-\frac{\alpha_{s}}{2}\frac{2\mu +1}{\sqrt{3(\mu^{2}+\mu +1)}}f_{s}$$ (9)

$$\mu =-\frac{\varphi +2}{2(\varphi +1)}$$ (10)

where *α*_*s*_ = *A*_*s*_/*A*_*c*_. *A*_*s*_ and *A*_*c*_ are the sectional areas of the steel tube and concrete, respectively. *F* = 0.16+0.53*α*, and *G* = 1.96−1.94*α*. *ϕ* is the hoop coefficient. *φ* = *αf*_*s*_/*f*_*ck*_. [*σ*_0_] is the ultimate stress of concrete.

#### Calculation of binding force

The circumferential strain of the steel tube is equal to the radial strain of the concrete, *ε*_*s*3_ = *ε*_*c*2_.


$$\frac{\sigma_{s3}}{E_{s}}=\frac{p_{s}}{E_{c}}-\frac{\mu_{c}}{E_{c}}(\sigma_{c1}+p_{s})$$ (11)

After sorting the above formulas, the following results are obtained.

$$p_{s}=\frac{\mu_{c}}{(1-\mu_{c})-\frac{r_{s}E_{c}}{t_{s}E_{s}}}\sigma_{c1}$$ (12)

Order $n=\frac{\mu_{c}}{(1-\mu_{c})-\frac{r_{s}E_{c}}{t_{s}E_{s}}}$

where *p*_*s*_ = *nσ*_*c*1_

### Initial stress analysis

The initial stress of the concrete is the force on the concrete when there is no creep in the reinforced concrete-filled steel tube columns. The steel tube exhibits a hoop restraint effect on the concrete; therefore, this effect should be considered in the initial stress solution.

$$N=N_{r}+N_{c}$$ (13)

$$N=\sigma_{r1}A_{r}+\sigma_{c1}A_{c}$$ (14)

where *N*_*r*_ and *N*_*c*_ are the axial forces of the longitudinal reinforcement and concrete, respectively. *A*_*r*_ and *A*_*c*_ are the sectional areas of the longitudinal reinforcement and concrete, respectively.

The longitudinal strain of the longitudinal reinforcement is equal to the longitudinal strain of concrete: *ε*_*r*1_ = *ε*_*c*1_.

The results are as follows:

$$\sigma_{r1}=(\frac{\sigma_{c1}}{E_{c}}-\frac{2\mu_{c}}{E_{c}}p_{1})E_{r}=[\frac{1}{E_{c}}-\frac{2\mu_{c}}{E_{c}}n]E_{r}\sigma_{c1}$$ (15)

$$N=[(\frac{1}{E_{c}}-\frac{2\mu_{c}}{E_{c}}n)A_{r}E_{r}+A_{c}]\sigma_{c1}$$ (16)

$$\sigma_{c1}=\frac{N}{(\frac{1}{E_{c}}-\frac{2\mu_{c}}{E_{c}}n)E_{r}A_{r}+A_{c}}$$ (17)

## Creep calculation formula

Concrete and steel tubes have different Poisson’s ratios. Under axial compression loading, the steel bar, concrete, and steel tube are in stress simultaneously. The steel tube exhibits a hoop restraint effect on concrete. Under the long-term action of the external load, the concrete will be in the unloading state owing to creep. The stress on the section of the composite columns is redistributed, and transferred to the longitudinal reinforcement and steel tube, thereby increasing the stress of the longitudinal reinforcement and steel tube.

Based on the creep characteristics of reinforced CFST columns, the creep theory of concrete under a multiaxial stress state proposed by Neville [26] was adopted. Under the condition of multiaxial stress, the creep function of concrete in the three directions can be expressed as follows:

$$c_{i}=\frac{c}{\sigma_{u}}[\sigma_{i}-\mu_{cp,i}(\sigma_{j}+\sigma_{k})]$$ (18)

where *c*_*i*_ is the net creep of concrete under multiaxial stress, *σ*_*u*_ is the stress on concrete under uniaxial stress, and *μ*_*cp*,*i*_ is the effective Poisson’s ratio in the *i* direction and is related to the state of action of *σ*_*i*_, *σ*_*j*_, and *σ*_*k*_. *σ*_*i*_, *σ*_*j*_, and *σ*_*k*_ are the stresses in the *i*, *j*, and *k* directions respectively. *c* is the creep of concrete under uniaxial stress. Because the concrete in the steel tube-reinforced concrete column is in the unloading state during creep, the creep theory for concrete creep is used to analyze the creep of the steel tube-reinforced concrete column.

$$c=[1.51(1-e^{‐{2.7(t‐t}_{0})})+3.34(1-e^{‐{0.14(t‐t}_{0})})+]2.17(1-e^{{‐1.15(t‐t}_{0})})+8.85(1-e^{{‐0.015(t‐t}_{0})})\times 10^{‐6}$$

where *t*_0_ is the loading age, and *t* is the time.

Han and Wang [18] derived the relationship with the ratio of the principal stress in the three directions from experimental data.


$$\mu_{cp,i}=0.160-0.074\times \frac{\sigma_{i}}{\sigma_{j}+\sigma_{k}}+0.028\times {\left(\frac{\sigma_{i}}{\sigma_{j}+\sigma_{k}}\right)}^{2}$$ (19)

The concrete is in the state of equal lateral stress: *σ*_*c*2_ = *σ*_*c*3_ = *p*_*s*_, *p*_*s*_ = *nσ*_*c*1_.

Therefore, the longitudinal effective Poisson’s ratio *μ*_*cp*,1_ is as follows:

$$\mu_{cp,1}=0.16-\frac{0.037}{n}+\frac{0.007}{n^{2}}$$ (20)

The longitudinal creep *c*_1_ can be expressed as $c_{1}=\frac{c}{\sigma_{u}}(\sigma_{c1}-2\mu_{cp,1}p_{s})$

The longitudinal stress of concrete *σ*_*c*1_ is equal to that of concrete under uniaxial stress *σ*_*u*_, i.e., *σ*_*u*_ = *σ*_*c*1_.

The longitudinal creep *c*_1_ can be reduced to

$$c_{1}=(1-2\mu_{cp,1}\cdot n)\cdot c$$ (21)

When creep occurs in the reinforced CFST columns, the stress on the section is redistributed; however, this redistribution does not affect the external load. Therefore,

$$N_{{}_{c}}^{c}+N_{r}^{c}=0$$ (22)

$$\sigma_{c1}^{c}A_{c}+\sigma_{r1}^{c}A_{r}=0$$ (23)

where $\sigma_{c1}^{c}$ and $\sigma_{r1}^{c}$ are the longitudinal stress increments of the concrete and steel bars, respectively.

The creep of concrete can be expressed as $\varepsilon_{c1}^{c}=(\sigma_{c0}+\sigma_{c1}^{c})\cdot c_{1}$.

The influence of creep on the transverse deformation of RCFST columns can be expressed as follows:

$$\varepsilon_{s3}^{c}=\frac{\mu_{s}}{E_{s}}\frac{r_{s}}{t_{s}}\Delta p_{s}$$ (24)

$$\varepsilon_{c2}^{c}=(p_{s}+\Delta p_{s})\cdot c_{2}$$ (25)

where *c*_2_ is the radial creep of concrete. Δ*p*_*s*_ is the increase in *p*_*s*_ due to the creep.

Because the increment in *p*_*s*_ is very small, it can be ignored; thus, the lateral creep of concrete can be expressed as $c_{2}=\frac{c}{\sigma_{u}}[p_{s}-\mu_{cp,2}(p_{s}+\sigma_{c1})]$, where *μ*_*cp*,2_ is the Poisson’s ratio of the lateral creep of concrete.

Order *σ*_*u*_ = *p*_*s*_, then

$$c_{2}=[1-\mu_{cp,2}(1+\frac{1}{n})]\cdot c$$ (26)

$$\mu_{cp,2}=0.16-\frac{0.074}{1+\frac{1}{n}}+\frac{0.028}{{\left(1+\frac{1}{n}\right)}^{2}}$$ (27)

Based on the compatibility condition of the radial deformation, the increment in the steel tube binding force was obtained.


$$\Delta p_{s}=\frac{n\cdot \sigma_{c0}c_{2}}{\frac{\mu_{s}}{E_{s}}\frac{r_{s}}{t_{s}}-c_{2}}$$ (28)

From the equal longitudinal deformation of the steel bar and concrete, we have $\varepsilon_{r1}^{c}=\varepsilon_{c1}^{c}$. In this case,

$$\sigma_{r1}^{c}=(\sigma_{c0}+\sigma_{c1}^{c})c_{1}E_{r}$$ (29)

The stress increment of the concrete under creep is as follows:

$$\sigma_{c1}^{c}=-\frac{c_{1}E_{r}A_{r}}{E_{r}A_{r}c_{1}+A_{c}}\sigma_{c0}$$ (30)

Thus, the creep formula for the composite columns under axial compression is derived:

$$\varepsilon_{sc}^{c}=[1-\frac{c_{1}E_{r}A_{r}}{E_{r}A_{r}c_{1}+A_{c}}]c_{1}\sigma_{c0}$$ (31)

## Influence of design parameters

### Formula validation

**Fig 4:**
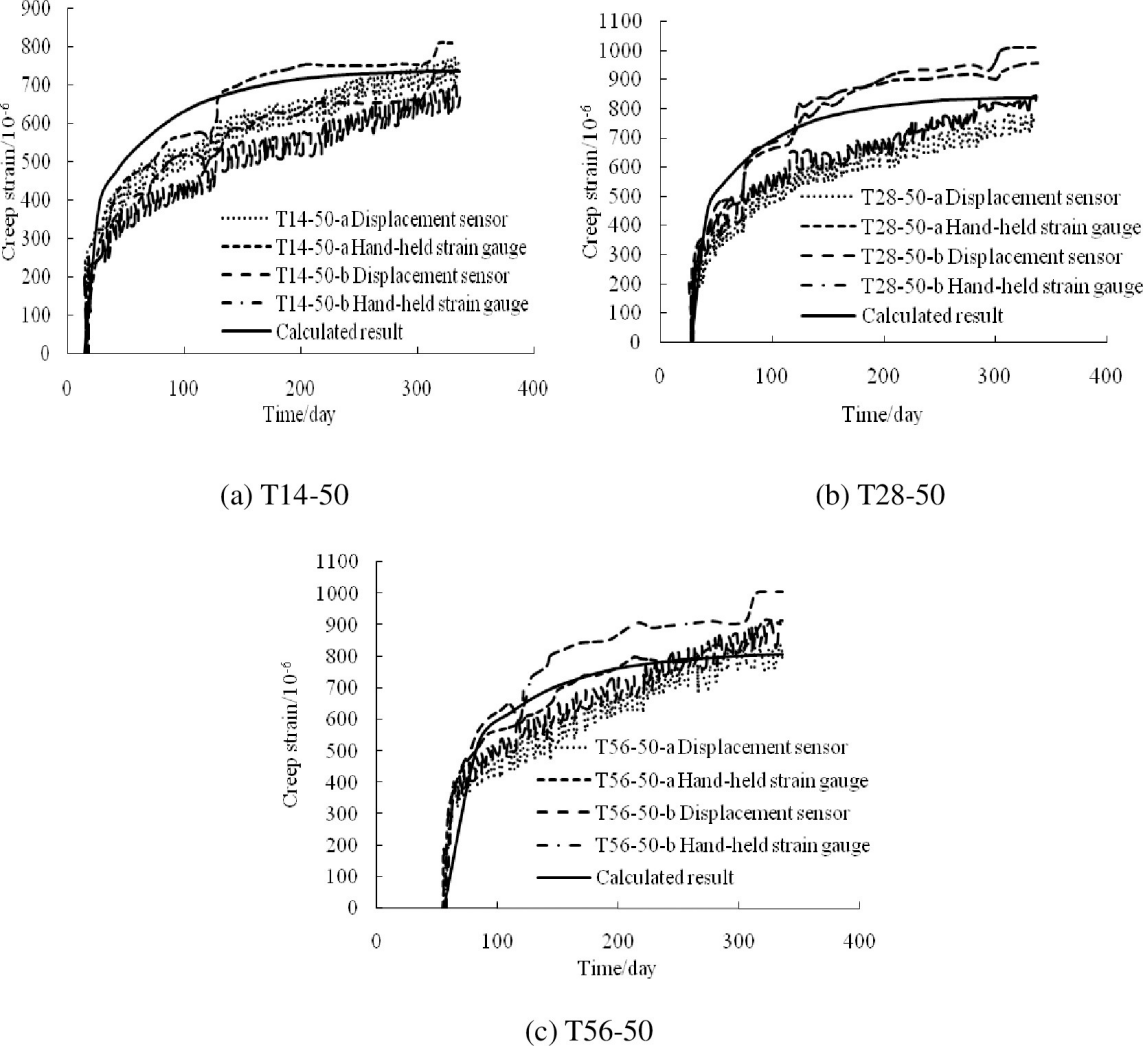
Comparison of the test results with the calculation results reported in [22].

Based on the above theory, a calculation and analysis program for the creep of RCFST columns under axial compression was developed. To verify its correctness, the calculated creep strain–time curves were compared with those of test specimens t14-50, t28-50, and t56-50 reported in [22], as shown in Fig 4. In [22], a displacement sensor and a hand-held strain gauge were used, and the error in the two methods was approximately 10%. The external diameter, wall thickness, steel content, yield strength, elastic modulus, and Poisson’s ratio of the steel tube were 219 mm, 2 mm, 3.6%, 373.5 MPa, 1.83×10^5^ MPa, and 0.248, respectively. Six HRB400 bars 18 mm in diameter were used as the longitudinal reinforcement, with a reinforcement ratio of 4%. The concrete grade was C50.

Fig 4(A) shows that the compressive strength of the concrete prism test block is 36.1 MPa, the elastic modulus is 2.92×10^4^ MPa, and the applied load is 653 kN. Fig 4(B) shows that the compressive strength of the concrete prism test block is 39.5 MPa, the elastic modulus is 3.05×10^4^ MPa, and the applied load is 725 kN. Fig 4(C) shows that the loading age is 56 days, the compressive strength of the concrete prism test block is 41.6 MPa, the elastic modulus is 3.55×10^4^ MPa, and the applied load is 784 kN. Fig 4 shows that the creep of the composite columns increases rapidly in the initial stages of loading (within 28 days), the growth rate is relatively low after 28 days, and it tends to stabilize after approximately six months. In general, the calculated results are in good agreement with the experimental results.

By comparing with the results reported in [22], the calculated results were found to be in good agreement with the experimental results under the conditions of different steel tube diameters, steel tube wall thicknesses, cross-sectional areas of the longitudinal reinforcement, concrete strengths, and action loads. This confirms the feasibility of the creep formula in analyzing the creep behavior of RCFST columns under long-term axial compression.

### Influence of design parameters

Based on the calculation results, the creep of the RCFST members under axial compression was analyzed. The basic parameters used in the calculation were as follows: the outer diameter, wall thickness, elastic modulus, yield strength, Poisson’s ratio, concrete grade, and elastic modulus of the steel tube were 165 mm, 1.5 mm, 1.88×10^5^ MPa, 345 MPa, 0.28, C50, and 3.45×10^4^ MPa, respectively. Six HRB400 bars 10 mm in diameter were used as the longitudinal reinforcement; the elastic modulus of the steel bar was 1.97×10^5^ MPa, the initial stress level of concrete *n*_*c*_ was 0.35. With the other parameters immutable, the effects of the stress level, wall thickness (content ratio), and reinforcement ratio on the creep behavior were studied.

#### Influence of stress level

**Fig 5:**
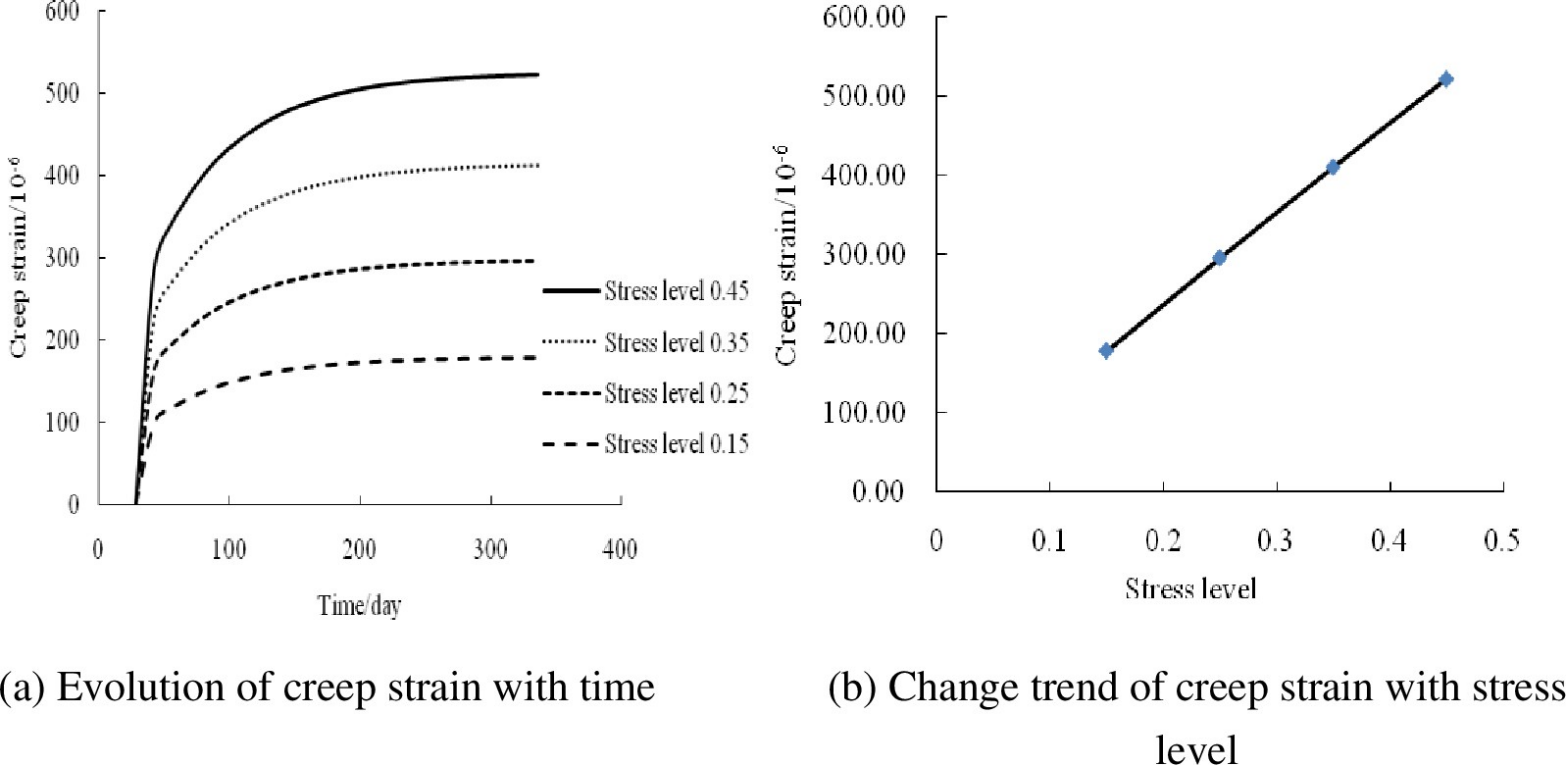
Influence of stress level.

The creep strain–time curves of RCFST columns under axial compression with stress levels of 0.45, 0.35, 0.25, and 0.15 were calculated and analyzed using the developed creep analysis program, as shown in Fig 5. The creep strain of the composite columns increases with the stress level under axial compression. This is because the concrete stress increases with the stress level, thereby increasing the creep strain of the composite columns. The creep strains at stress levels of 0.25, 0.35, and 0.45 were 65.9%, 129.6%, and 191.2% higher than that at a stress level of 0.15, respectively. The creep strain of the composite columns increased linearly with the increase in the stress level.

#### Influence of steel tube wall thickness (steel ratio)

**Fig 6:**
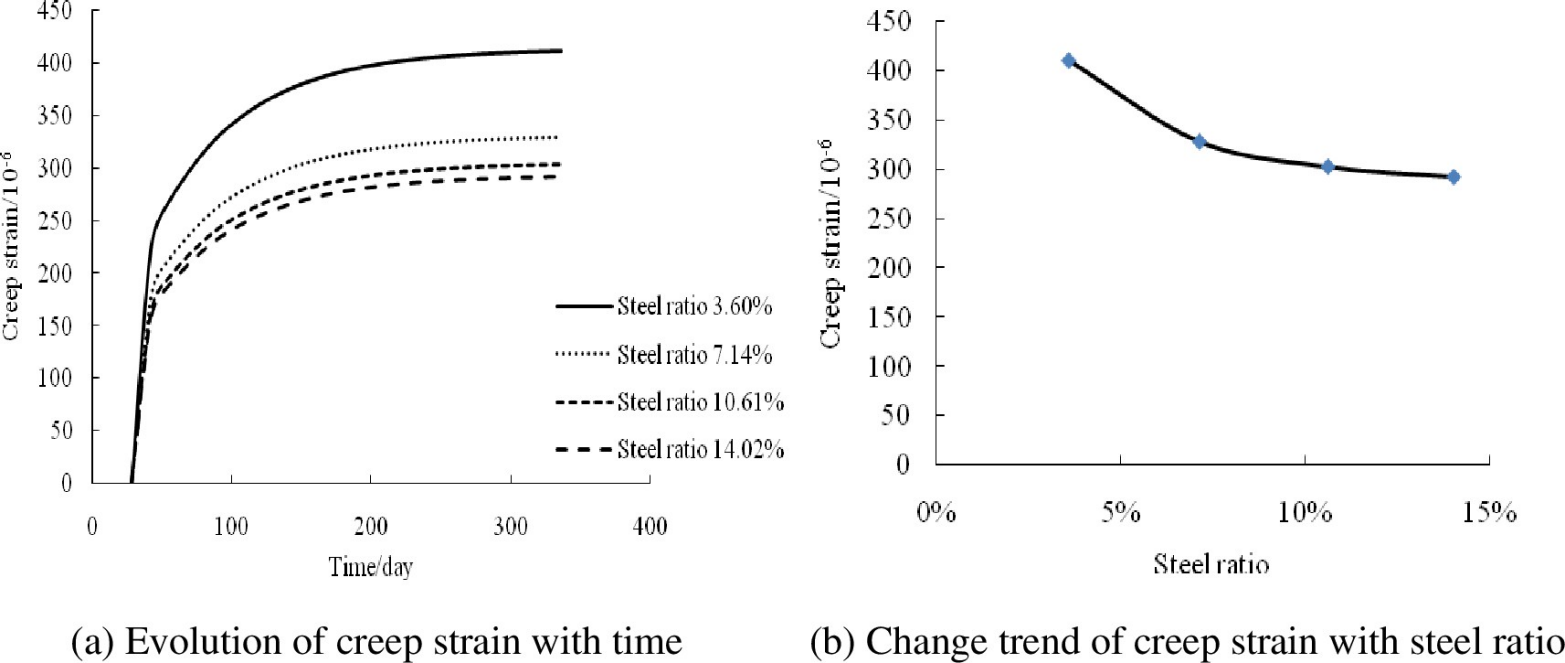
Influence of steel tube wall thickness (steel ratio).

Based on the creep analysis program, the creep strain–time curves for steel tube wall thicknesses of 6, 4.5, 3, and 1.5 mm—i.e., steel ratios of 14.02%, 10.61%, 7.14%, and 3.60%—were calculated and analyzed, as shown in Fig 6. The creep strain of the composite columns decreased with the increase in the steel content under axial compression. This is because with the increase in the steel content, the binding force of the steel tube increases and the effect of concrete decreases, thereby reducing the creep strain of the composite columns. The creep strains at steel ratios of 7.14%, 10.61%, and 14.02% were 20.1%, 26.4%, and 28.9% lower than that at a steel ratio of 3.60%, respectively. The creep strain of the composite columns did not change linearly with the increase in the steel content. When the steel content increased to a certain extent, its effect on the creep deformation decreased.

#### Influence of reinforcement ratio

**Fig 7:**
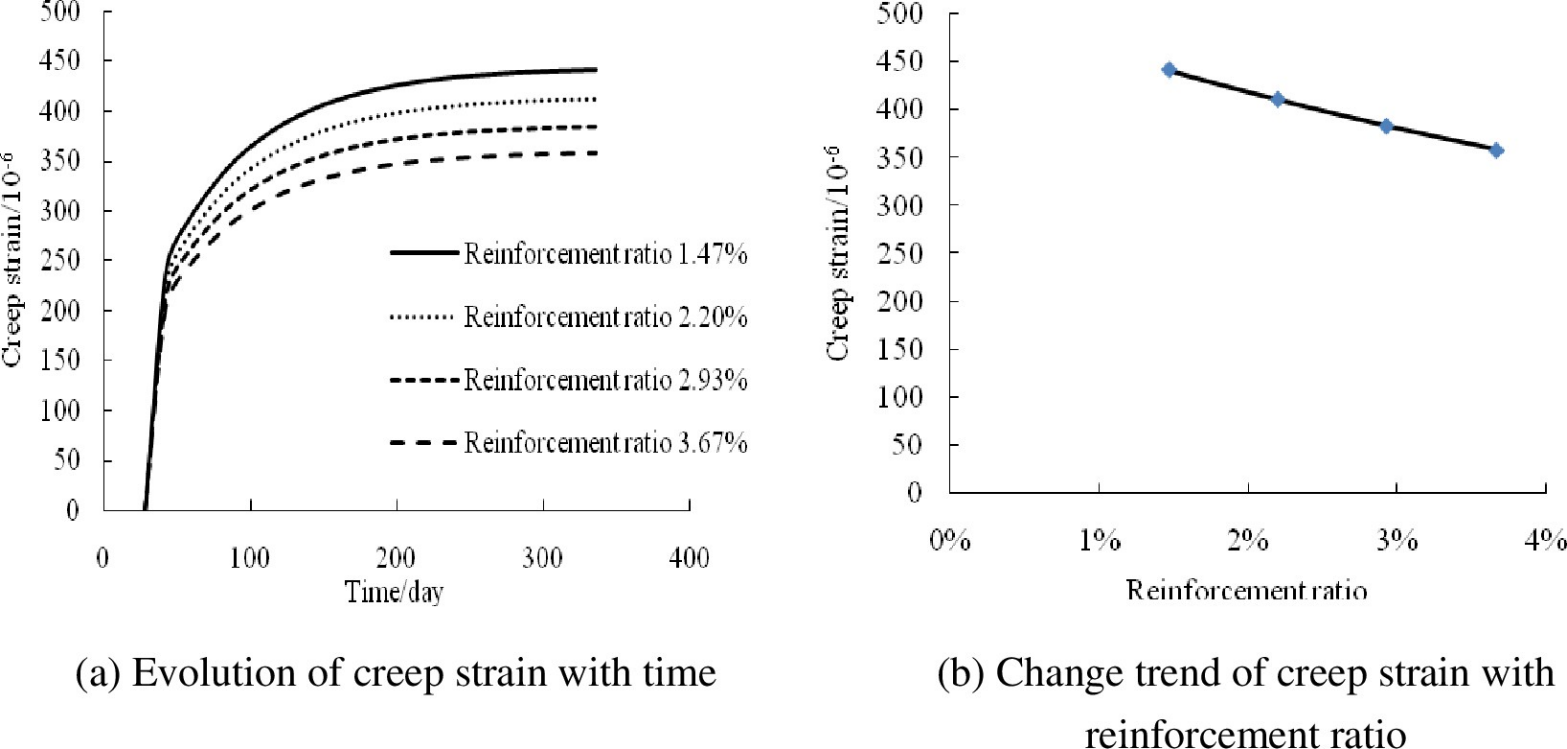
Influence of reinforcement ratio.

Based on the creep analysis program, the creep strain–time curves for reinforcement ratios of 1.47%, 2.20%, 2.93%, and 3.67% were calculated and analyzed, as shown in Fig 7. The creep strain of the composite columns under axial compression decreased with the increase in the reinforcement ratio. This is because with the increase in the reinforcement ratio, the effect of concrete decreases, thereby reducing the creep strain of the composite columns. The creep strains at reinforcement ratios of 2.20%, 2.93%, and 3.67% were 6.92%, 13.18%, and 18.85% lower than that at a reinforcement ratio of 1.47%. The creep strain of the composite columns decreased linearly with the increase in the reinforcement ratio.

The above analysis shows that the creep strain increases with the increase in the stress level. When the stress level ranges from 0.15 to 0.45, the creep strain increases by 191.2%, whereas it decreases with the increase in the steel content. When the wall thickness ranges from 1.5 to 6 mm and the steel content ranges from 3.60% to 14.02%, the creep strain is reduced by 28.9%. In comparison, it decreases with the increase in the reinforcement ratio. When the reinforcement ratio ranges from 1.47% to 3.67%, the creep strain is reduced by 18.85%. The stress level had a significant influence on the creep of the composite columns under axial compression, followed by the steel ratio. The influence of the reinforcement ratio on the creep was relatively small.

## Conclusions

In this study, based on the mechanical characteristics of reinforced concrete-filled steel tubular columns under axial compression, a creep calculation formula for composite columns under axial compression was established on the basis of the multiaxial stress and flow theory of concrete.

With the established formula, a creep analysis program was developed, and the creep strain–time curve was derived and analyzed. The results showed that the creep of the composite columns increases rapidly in the initial stages of loading (within 28 days). The growth rate was relatively low after 28 days and tended to stabilize after approximately six months. The creep program was verified by existing tests.

On this basis, the influence of the main design parameters on the axial compression creep of the composite columns was determined and analyzed. The stress level had a significant influence on the creep of composite columns under axial compression, followed by the thickness of the steel tube wall (steel ratio). The influence of reinforcement ratio had a relatively small influence on the creep.

This study only analyzed the creep characteristics of RCFST columns in the elastic working range; the creep problems in the elastoplastic and plastic stages need to be further studied.

## Supporting information

S1 Data (XLS)
